# Loss of Zebrafish *lgi1b* Leads to Hydrocephalus and Sensitization to Pentylenetetrazol Induced Seizure-Like Behavior

**DOI:** 10.1371/journal.pone.0024596

**Published:** 2011-09-16

**Authors:** Yong Teng, Xiayang Xie, Steven Walker, Meera Saxena, David J. Kozlowski, Jeff S. Mumm, John K. Cowell

**Affiliations:** 1 GHSU Cancer Center, School of Medicine, Georgia Health Sciences University, Augusta, Georgia, United States of America; 2 Department of Cellular Biology and Anatomy, School of Medicine, Georgia Health Sciences University, Augusta, Georgia, United States of America; 3 Vision Discovery Institute, School of Medicine, Georgia Health Sciences University, Augusta, Georgia, United States of America; 4 Program in Developmental Neurobiology, Institute of Molecular Medicine and Genetics, School of Medicine, Georgia Health Sciences University, Augusta, Georgia, United States of America; 5 Luminomics Inc, Augusta, Georgia, United States of America; Ecole Normale Supérieure de Lyon, France

## Abstract

Mutations in the LGI1 gene predispose to a hereditary epilepsy syndrome and is the first gene associated with this disease which does not encode an ion channel protein. In zebrafish, there are two paralogs of the LGI1 gene, *lgi1a* and *lgi1b*. Knockdown of *lgi1a* results in a seizure-like hyperactivity phenotype with associated developmental abnormalities characterized by cellular loss in the eyes and brain. We have now generated knockdown morphants for the lgi1b gene which also show developmental abnormalities but do not show a seizure-like behavior. Instead, the most striking phenotype involves significant enlargement of the ventricles (hydrocephalus). As shown for the lgi1a morphants, however, lgi1b morphants are also sensitized to PTZ-induced hyperactivity. The different phenotypes between the two lgi1 morphants support a subfunctionalization model for the two paralogs.

## Introduction

Mutations in the Leucine-rich, glioma inactivated 1 (LGI1) gene predispose to a hereditary form of epilepsy [1] known as autosomal dominant partial epilepsy with auditory features (ADPEAF). This disease is characterized by partial temporal lobe seizures accompanied by acoustic auras with an onset between 8–50 years of age [2]. The LGI1 gene [3], carries four and a half tandem repeats of a leucine rich repeat (LRR) motif at the N-terminal end and is a secreted protein [4]–[6]. LGI1 receptors that have been defined so far, and depending on the cell context, are the disintegrin and metalloprotease (ADAM) members 22 and 23 [7]–[9] and the Nogo receptor 1 [10]. The ADAM 22/23 molecules do not carry metalloproteinase domains and appear to be implicated in cell adhesion [8], [11]–[12], which is consistent with the suggestion that LGI1 influences cell movement and invasion through reorganization of the actin cytoskeleton [13]–[15]. Thus, LGI1 is the first epilepsy predisposing gene that does not encode a structural component of an ion channel.

LGI1 mutant null mice display a seizure phenotype, with early onset between 10–21 days after birth [16]–[18]. Electroencephalography (EEG) recordings in live mutant null mice indicated seizures originating in the hippocampus [Charbol et al 2010]. Electrophysiology analysis of CA1 hippocampal neurons suggests differing mechanisms, with Yu et al [18] describing a hyper excitability involving excess glutamate release from the presynaptic membrane, compared with the suggestion by Fukata and colleagues that LGI1 reduces AMPAR-mediated synaptic currents in the hippocampus [17]. The mechanism suggested by Yu at al [18] was supported by the studies in a BAC transgenic mouse expressing a truncated form of LGI1 anticipated to act as a dominant/negative [19] which show abnormal dendritic pruning of hippocampal neurons. Recently it has been shown that limbic encephalitis results from an auto immunity to the LGI1 protein [20], which results in epileptic seizures, memory loss and confusion as well as generalized encephalitis in these patients. Thus, although the role of LGI1 in seizure development is now well established [21], the underlying molecular mechanisms behind this phenotype are still largely unknown.

With the intent of developing a more tractable, vertebrate model to study the function of LGI1, we used morpholino knockdown strategies to inactivate LGI1 orthologs in developing zebrafish embryos [22]. The lgi1a morphant fish show a distinct seizure-like behavior which was similar to that induced as a result of treatment with epilepsy-inducing drugs [23]. The lgi1a knockdown fish also showed developmental abnormalities, including abnormal tail shape, smaller eyes and reduced brain mass accompanied by increased apoptosis [22]. Evidence for abnormal brain development has also been suggested in imaging studies of ADPEAF patients [24]–[25], possibly indicating a role for LGI1 in brain development. These observations are consistent with those from gene expression studies using cell culture systems implicating LGI1 in axon guidance pathways [15]. The zebrafish knock down model, therefore, provides a potentially valuable model to study the role of LGI1 in early development of the brain and its relationship with the underlying mechanism of seizure induction.

The zebrafish genome has undergone a partial duplication during evolution, resulting in two different paralogs for many mammalian genes [26]. The LGI1 gene was part of that duplication, generating the zebrafish *lgi1a* and *lgi1b* genes. In situ hybridization analysis of lgi1a/b gene expression [27] demonstrated a distinct, albeit overlapping, expression pattern for each homolog, suggesting a concomitant subfunctionalization. The seizure-like phenotype and developmental abnormalities described by Teng et al [22], resulted from the knockdown of the *lgi1a* gene. We have now generated knockdown morphants for the *lgi1b* gene which, consistent with the suggested subfunctionalization of these genes, demonstrate a very different phenotype. These morphants do not develop the overt seizure-like behavior seen in the lgi1a morphants. The lgi1b morphants, however, display a hypersensitivity to the epilepsy-inducing drug PTZ, as also shown for the lgi1a morphants [22]. The lgi1b morphants also showed delayed overall development and smaller eyes and brains, as seen in the lgi1a morphants, with associated increased apoptosis. The main difference in gross phenotype involved the significantly increased ventricle size in the lgi1b morphants. Thus, the different phenotypes seen in the lgi1a and lgi1b morphants provides the opportunity to dissect the function of the lgi1 paralogs in zebrafish.

## Materials and Methods

### Zebrafish maintenance and stocks

Embryos from the wildtype Tü zebrafish strain were raised at 28.5°C. Embryos from natural matings were staged as described by Kimmel et al. [28] and kept in 1-phenyl-2-thiourea (PTU; 0.003%, Sigma, USA) to prevent pigment formation allowing for better confocal analysis. All animal protocols used in this study were reviewed and approved by the Medical College of Georgia's Institutional Animal Care and Use Committee; Protocol # BR10-12-391.

### Cloning and analysis of zebrafish lgi1b

For confirmation of the *lgi1b* sequence, the genomic DNA was extracted from the Tü strain of fish maintained in the MCG core facility. Primers were designed using the existing database sequence to amplify the genomic regions containing the start codon and the splice junctions for the first six exons of *lgi1b* which contain the leucine rich repeats. We generated PCR products for each region and cloned them into the TA vector (Invitrogen) for sequencing. Predicted protein sequence for the lgi1a (Accession #CAP08004.1) and lgi1b (Accession # AAI63593) of zebrafish and other species were obtained from GenBank. ClustalW alignments (http://www.ch.embnet.org/software/ClustalW.html) were prepared to compare the identity of the cloned sequence against previously identified human and mouse paralogs.

### Antisense depletion of Lgi1b

**Figure 1:**
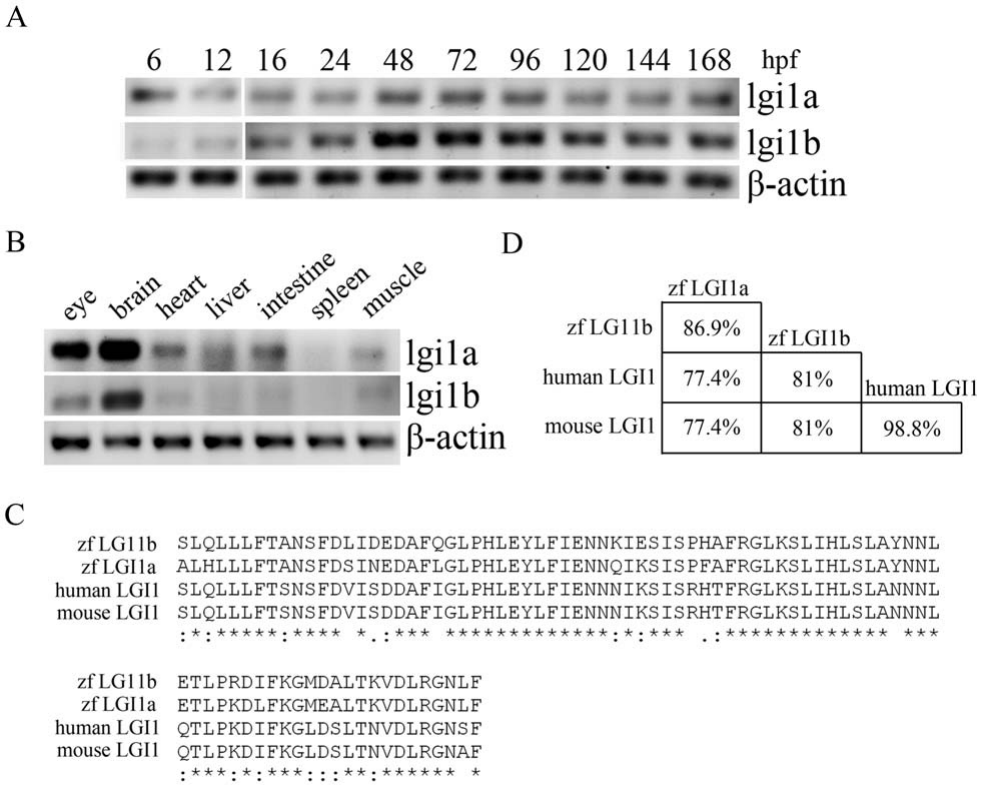
Expression patterns of *lgi1b* in zebrafish. Semi-qRT-PCR analysis of lgi1 expression levels at different developmental ages (A) or in different zebrafish tissues (B). (C) Significant identity between the zebrafish lgi1a and lgi1b and the human and mouse genes is apparent based on the amino acid sequences within the leucine-rich repeat motif (LRR). The asterisk indicates amino acids conserved in all species. (D) The percentage comparison of identical amino acids.

An *lgi1b*-specific morpholino antisense oligo (MO-E2, 5′-AACACTAAGGGAAACTCACAGAAGC-3′) targets the splice donor region of the intron between Exon2 and Exon3 and a 5-base mismatch control oligos (MO-E2mis, 5′- AACAgTAAcGcAAACTCAgAcAAGC-3′, mismatches in lower case) were obtained from Gene Tools (Philomath, OR, USA). The impact of the splice blocking MO was determined by RT-PCR with RNAs extracted from 24 to 72 hours post fertilization (hpf) embryos using the following primers (Figure 1A): p1-5′- AACAGGAGCATAAGAGCATA-3′; p2-5′- AATCCAGACTTCACAAACGA -3′; p3-5′- TCAAGTCAAAAGAATTGGCT-3′. For generation of the lgi1 double morphants, the morpholino targeting the E3/I3 junction in *lgi1a* (lgi1a MO-E3 [22], was used for co-injection with the lgi1b MO-E2.

### Rescue mRNA injections

The full-length zebrafish *lgi1b* cDNA was subcloned into the pCS2^+^ vector using *EcoRI/XbaI* sites and mRNA was synthesized *in vitro* using the mMessage mMachine SP6 Kit (Ambion, Austin, TX, USA). For MO rescue experiments, 20–60 pg of capped RNA was co-injected with 2 ng MO-E2 at one-cell-stage zygotes.

### Semi-quantitative RT-PCR (semi-qRT-PCR) and quantitative real-time RT-PCR (qRT-PCR) analysis

Ten embryos per group were collected at defined developmental stages. Total RNA was extracted from embryos using Trizol (Invitrogen, Carlsbad, CA, USA) and contaminating DNA was depleted using RNase-free DNase. Reverse-transcribed cDNA was analyzed using semi-qRT-PCR or qRT-PCR for target mRNA levels. For qRT-PCR assay, samples were amplified in triplicate using the BioRad iQ SYBR Green Supermix (BioRad, Hercules, CA, USA) on a BioRad iCycler equipped with an iCycler iQ Detection System. In addition to the *lgi1b* primers (p1 and p2) described above, the following primers were also used: *lgi1a* Forward 5′- ATCATTCGTCAAATCCGGCT-3′ and *lgi1a* Reverse 5′- AGATACTCCAGATGAGGGAG-3′; *lgi2a* Forward 5′- TGCTGGATGTGAATAAACGT-3′ and *lgi2a* Reverse 5′- AGTAACTGTAGGGAGGGCAT-3′, *lgi2b* Forward 5′- TTACACTCCGCTTTAAAACC-3′ and *lgi2b* Reverse 5′-AACTAAGAAGGAGCAACTGC-3′, *lgi3* Forward 5′- GATCTTCTGCTATTGAGGTT-3′ and *lgi3* Reverse 5′- TAGTCAGAGAGATGATTCCC-3′; *c-fos* Forward 5′- CCAAAACAGAGAAAAGAGCA-3′ and *c-fos* Reverse 5′- TCGGGTTGTAGGATTGAGCT-3′; *elfα* Forward 5′- CTTCTCAGGCTGACTGTGC-3′ and *elfα* Reverse 5′- CCGCTAGCATTACCCTCC-3′; *β-actin* Forward 5′- CGAGCAGGAGATGGGAACC-3′ and *β-actin* Reverse 5′- CAACGGAAACGCTCATTGC-3′. Relative levels of target mRNA expression were calculated using the 2^−ΔΔCT^ method and *β-actin* was used for normalization.

### Acridine orange staining and BrdU treatment

Live embryos were dechorionated manually and stained with 0.1 µg/ml acridine orange at room temperature. After 10 min shaking, embryos were rinsed four times for 10 min each with 10% Hanks' saline solution (HSS) and live mounted for imaging. Samples were viewed using either a conventional fluorescence microscope or a confocal microscope. For cell proliferation assays, embryos were dechorionated and placed in 0.3× Danieau's solution (pH 6.8) on ice for 15 min, followed by 20 min incubation on ice in 10 mM 5-Bromo-2′-Deoxyuridine (BrdU, Sigma). Embryos were then placed in prewarmed embryo media for 3 min at 28.5°C, followed by fixation in 4% paraformaldehyde (PFA, Sigma) for 2 h at room temperature and dehydrated in 100% methanol at −20°C. Prior to anti-BrdU antibody staining, embryos were rehydrated and treated with proteinase K (10 µg/ml, Promega, USA) for 15 min, then fixed in 4% PFA for 30 min. After rinsing in PBST, embryos were treated with 2N HCl (Sigma, USA) for 1 h at room temperature and then incubated with 1∶500 BrdU antibody (Sigma, USA) overnight at 4°C. Following 4 rinses in PBST, embryos were incubated with a goat anti-mouse FITC secondary antibody for 30 min. Images were recorded using a confocal.

### Whole mount TUNEL assays

For TUNEL assays, embryos were staged and fixed overnight in 4% PFA in PBS at 4°C, then washed in PBS/0.1% Tween 20 (PBST), dechorionated, dehydrated stepwise in methanol, and stored at −20°C. After rehydration, embryos were incubated in 10 µg/ml proteinase K for 5 min at room temperature and re-fixed in buffered 4% PFA, followed by 4 rinses in PBST. Fragmented genomic DNA was identified using DeadEnd™ Colorimetric TUNEL System (Promega, USA) according to the manufacturer's instructions. Images were recorded using a confocal microscope.

### In vivo confocal microscopy and image processing

Confocal images were captured using an upright confocal laser-scanning microscope (FV1000; Olympus, Tokyo, Japan). All live imaging was performed as previously described [22]. Confocal stacks were further processed using ImageJ 1.41 (NIH, USA). In some cases, z-projections using several slices were constructed; in others, single representative slices were selected. In all figures, comparisons are made between images that were processed equivalently.

### Drug treatment, behavioral monitoring and statistical analysis

Representative single larvae were separated into individual wells of a 48-well plate in a semi-randomized pattern such that each group was evenly distributed. Plates were then placed inside a ZebraBox, and monitored using the ZebraLab video activity monitoring system (ViewPoint Life Sciences, Montreal, Canada). Fish motility was quantified before treatment for 30 min to obtain an activity baseline and then after pentylenetetrazole (PTZ, 2.5 mM, Sigma, USA) treatment for 2 hrs. Measurements of fish activity and the statistical analysis of Viewpoint activity data was processed using Matlab 7.6 and the statistical software R as described previously [Teng et al 2010].

### Western blotting analysis

For each experiment, 15 embryos were decapitated using Dumont #55 forceps (World Precision Instrument, Sarasota, FL) and pooled for protein extraction using NP40 lysis buffer with protease inhibitors as described previously [29]. Western blotting analysis was undertaken by incubation with either an anti-c-Fos antibody (Santa Cruz Biotechnology, CA) or an anti-β-actin antibody (Sigma, MO), at 4°C overnight. After washing, membranes were incubated with HRP-conjugated secondary antibody (Pierce, IL) for 1 h at room temperature. Detection of the target proteins was completed with Supersignal West Pico chemiluminescent substrate (Pierce, Rockford, IL).

## Results

### Developmental expression of *lgi1* genes in zebrafish

Differential patterns of mRNA expression suggest subfunctionalization of zebrafish paralogs. To compare the expression profile of the zebrafish *lgi1* genes, we undertook RT-PCR analysis (Figure 1A) using RNA derived from a pool of 10 wildtype zebrafish during early development (6–168 hpf). This analysis demonstrated that lgi1a mRNA levels were detectable as early as 6 hpf, while the lgi1b expression was first detected at 12 hpf. Strikingly, both genes show robust expression during 48–96 hpf and then decreased to lower levels which were sustained over the first 7 dpf. These observations support the suggestion that both genes may play important, albeit possibly different, roles in embryogenesis. When the expression pattern of the *lgi1a* and *lgi1b* genes was analyzed in 1-month-old fish, lgi1b showed a more restricted expression pattern, being largely confined to the eye and brain, with lgi1a expression also seen in the heart, intestine and liver (Figure 1B).

### Molecular consequences of lgia1b knockdown

**Figure 2:**
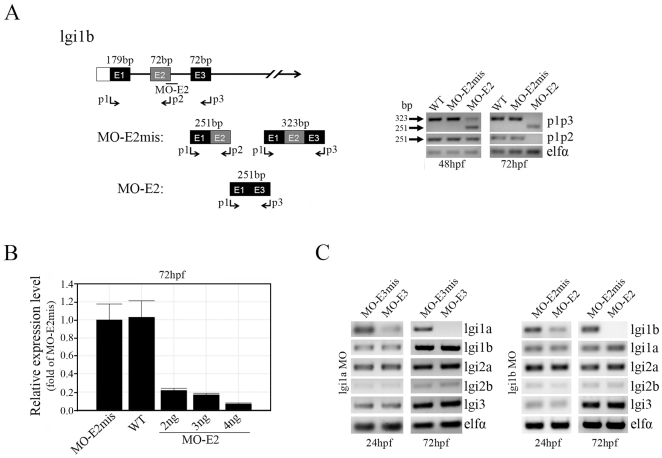
Splice-site-targeted morpholino oligonucleotides alter normal lgi1b splicing. (A) A schematic diagram of the partial pre-mRNA map showing the predicted outcomes in lgi1b morphants (left panel). The exons are shown in boxes, labeled with the corresponding exon number E1–E3, and the introns are represented by solid lines. The location of the exon 2 splice site, targeted by the MO-E2 morpholino, and the primer binding sites (p1, p2, p3, arrows) for PCR validation are indicated. Semi-qRT-PCR analysis (right panel) of lgi1b transcript levels in wild-type fish, MO-E2mis and MO-E2 morphants at different time points (48 and 72 hpf) show aberrant splicing in the MO-E2 (2 ng) morphants which generates an abnormal (251 bp) mRNA (lacking exon 2) using primers p1/p3. (B) qRT-PCR analysis of lgi1b transcript levels in wild-type, MO-E2mis and MO-E2 morphants following injection of three different concentrations of MO-E2 (2, 3 and 4 ng). (C) Semi-qRT-PCR analysis of transcript levels of the zebrafish lgi1 family members after knockdown of lgi1a (left) and lgi1b (right) respectively.

Comparison of the zebrafish lgi1a and lgi1b amino acid sequence with human and mouse proteins demonstrate high (63% and 67% respectively) homology [22]. Over the LRR, in particular, there is even higher identity (77% and 80% respectively) suggesting a highly conserved function for this motif (Figure 1C and D). Before designing the targeting MO for lgi1b, we resequenced its exon-intron junctions in the Tü strain available in our laboratory, which was largely consistent with the reference sequence. Our derived sequence was then used to determine that the optimal MO design for successful targeting of *lgi1b* at the E2/I2 junction (MO-E2) which lies within the highly conserved LRR. A five base pair *lgi1b* mismatch MO (MO-E2mis) was used as a control in all experiments. Targeting the E2/I2 junction in *lgi1b* was expected to result in exon skipping, leading to a smaller mRNA lacking exon 2, which was confirmed using the PCR strategy outlined in Figure 2. Using semi-qRT-PCR analysis of RNA extracted from 24–72 hpf embryos, primers p1/p2 which were designed to amplify between exons 1 and 2 and primers p1/p3 which amplify between exons 1 and 3, we demonstrated that MO-E2 treatment resulted in the deletion of exon 2. As a result, a 251 bp fragment was generated using primers p1/p2 in the lgi1b morphants compared with a 323 bp fragment in the control fish (wildtype and MO-E2mis morphants). After 72 hpf, l*gi1b* mRNA levels were almost completely absent (Figure 2), which is the time at which expression of *lgi1b* is normally maximal (Figure 1). To determine the efficiency of knockdown, varying concentrations of MO-E2 were injected into one-cell stage embryos (2, 3 and 4 ng) and qRT-PCR was performed to quantify *lgi1b* mRNA levels at 72 hpf. These studies demonstrated a dose-dependent reduction of lgi1b mRNA levels. Approximately 80% knockdown was achieved using 2 or 3 ng and more than 90% knockdown was seen using 4 ng. Unfortunatley, the highest dose (4 ng) leads to severe abnormal development and significant premature death (∼62% mortality). The specificity of the lgi1b MO for its target is shown in Figure 2, where it is clear that knockdown of lgi1b does not affect lgi1a mRNA levels and vice versa. In humans, the LGI gene family consists of four members [27], but in fish homology to only three of these genes have been described. There are two *lgi2* paralogs and only a single gene for lgi3. Knockdown of either lgi1a or lgi1b does not affect expression of any of the other zebrafish lgi family members (Figure 2).

### Knockdown of lgi1b leads to severe hydrocephalus and developmental brain defects

**Figure 3:**
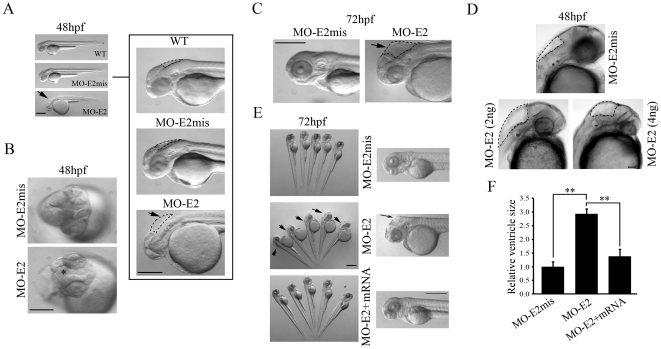
Morphological changes in 2 ng lgi1b morphants after 48 and 72 hpf. Compared with wild-type (WT) and MO-E2mis-injected embryos (A, C: lateral view and B: dorsal view), lgi1b morphants showed abnormal head development involving smaller eyes and overt hydrocephalus (arrows) in midbrain and hindbrain ventricles compared with wild-type and control morphants. These phenotypes are more obvious at 72 hpf. (D) Embryos were examined at 48 hpf after injection of two doses of MO-E2 (2 ng and 4 ng) or 4 ng of MO-E2mis. More severe hydrocephalus (demarcated by the dotted frames) and smaller eyes were seen in the high dose (4 ng) lgi1b morphants. (E) The consistent gross head and eye defects and hydrocephalus (left; arrows) in 72 hpf lgi1b morphants were rescued by co-injection of the full length lgi1b mRNA (lateral view), shown in more detail on the right. (F) Comparison of relative ventricle sizes in midbrain and hindbrain based on measurements from 10 embryos per group. ** = *p*<0.01. Scale bars: A, C and E = 500 µm; B = 50 µm; D = 25 µm.

**Figure 4:**
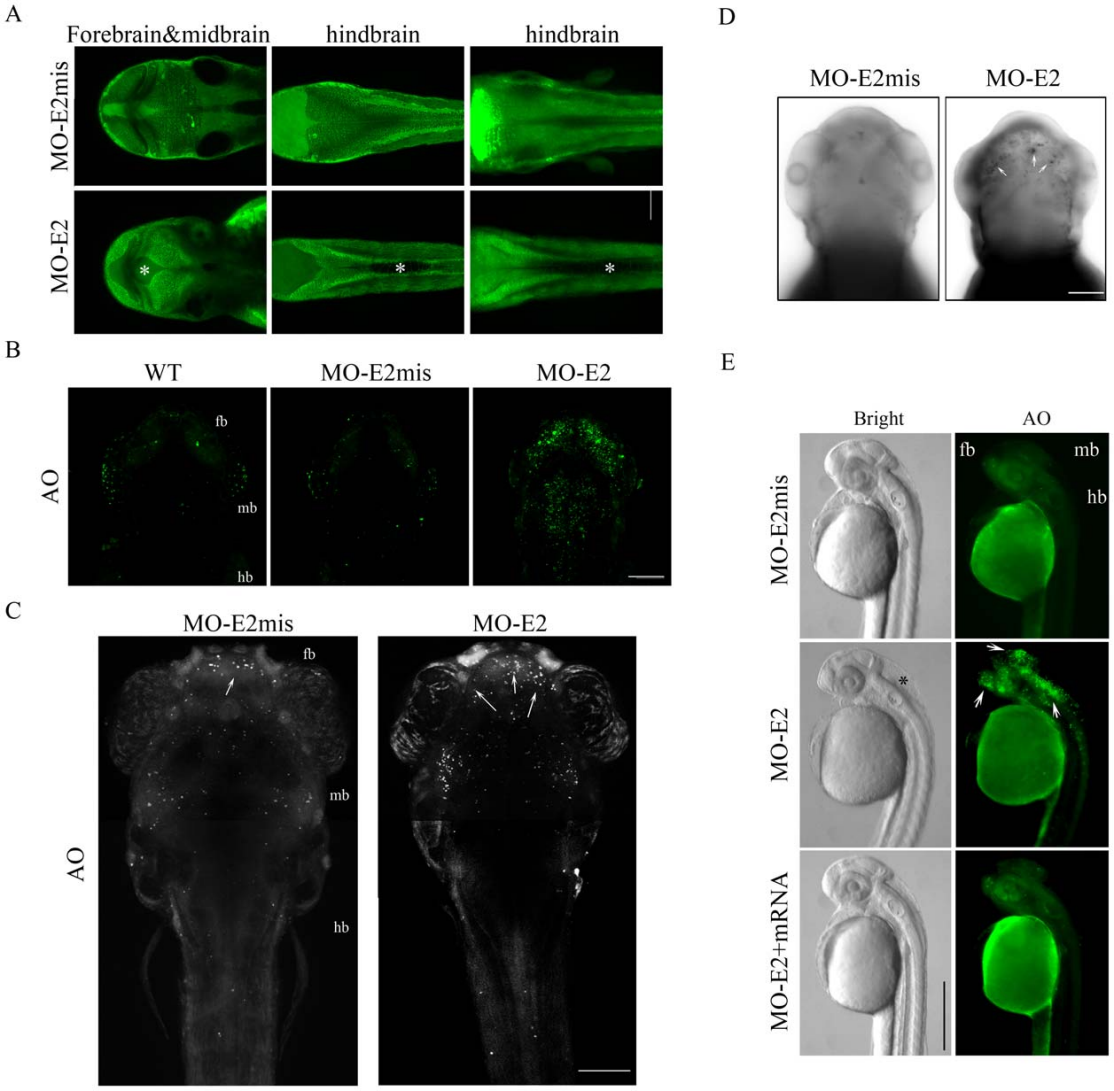
Confocal imaging analysis of hydrocephalus and apoptosis in lgi1b morphants. Representative single z-plane images (A) from (left) forebrain and midbrain, (center) hindbrain and (right) Z-stack images show MO-E2 morphants have enlarged ventricles (indicated by *) in both forebrain/midbrain and midbrain/hindbrain compared with mismatch controls (MO-E2mis). Scale bar = 50 µm. (B) Uninjected (WT), MO-E2mis and MO-E2 embryos stained with AO after 48 hpf showed increased cell death (green cells). (C) AO staining analysis showed increased apoptotic cells (White dots) could be detected in the forebrain of the lgi1b morphants after 72 hpf. (D) Whole mount TUNEL analysis further supports increased apoptosis in the forebrain at 72 hpf in lgi1b morphants. Black dots (white arrows) indicate TUNEL-positive cells. (E) AO staining shows apoptosis (arrows) was significantly reduced in lgi1b morphants co-injected with lgi1b mRNA at 36 hpf. (B–D) dorsal view, Z-stack confocal images. (E) a single lateral view image. Scale bars: B, C and D, 100 µm; E, 500 µm.

Abnormal phenotypes and behaviors in morphants is typically related to the dose of the MO administered [22] where, for lgi1a morphants for example, 3–4 ng MO produced seizure-like behavior but 2 ng did not. Similarly, other phenotypes affecting development of the brain and eyes were only detected at doses >2 ng [22]. In our analysis of the *lgi1b* gene, we used the same 2–4 ng range of MO concentrations. Control embryos were always injected with 4 ng of MO-E2mis. Close observation of the lgi1b morphants did not show an obvious seizure-like phenotype, even at the highest MO concentration (4 ng), which resulted in hyperactivity in the lgi1a morphants [22]. Instead, the lgi1b morphants (4 ng) showed abnormal development of the head, smaller eyes, severe edema of the heart and a noticable trunk curvature (Figure S1), which were seen in the lgi1a morphants [22]. The most striking phenotype in the lgi1b morphants, involved pronounced hydrocephalus in the midbrain and hindbrain ventricles compared with wild-type and control morphants (Figure S1), which was present even in the 2 ng morphants (Figure 3A, B and D). This phenotype could be observed as early as 24 hpf and became more obvious at 48 hpf and, at 3 dpf, this phenotype was evident in almost all lgi1b morphants (Figure 3C and E). Compared with the lower dose morphants (2–3 ng), high dose lgi1b morphants (4 ng) have more significant hydrocephalus, smaller eyes (Figure 3D) and high mortality within 3 days. Similarly, when followed for up to 10 days, only 8% of the 2 ng MO-E2 injected fish (n = 100) survived. Significant hydrocephalus, small eyes and severe heart edema were observed after 5 dpf, even though lgi1b expression levels had recovered by this time (Figure S2). These observations suggest that loss of lgi1b during early development produces permanent phenotypic changes. Taken together, these data indicate the effect of lgi1b MO on brain development was dose-dependent, and that higher doses of the morpholino increased the fraction of zebrafish with the morphant phenotype. Morphological changes of the brain in embryos treated with 2 ng of MO-E2 were further characterized using a live imaging method after staining with BODIPY (Figure 4). These confocal images show dilated ventricles, cell loss and reduced brain size, which suggests that lgi1b is required for normal brain development in zebrafish.

To further demonstrate the specificity of the MO targeting strategy, we performed RNA-rescue assays. The 2 ng of MO-E2 was co-injected with 60 pg of a full length, mature lgi1b mRNA. In this case, more than 71% of the embryos (n = 120) appeared morphologically similar to the wild type and mismatch morphants with normal appearing ventricles and normal development of the eyes and brains (Figure 3E), while injection of lgi1b mRNA alone did not result in phenotypical alterations (data not shown). At 48 hpf, the lgi1b knockdown morphants show a significant increase in the size of hindbrain ventricle compared with both mismatch and rescue morphants (p<0.01). These results suggest that the phenotype is due to inhibition of lgi1b expression.

### Loss of *lgi1b* leads to increased cell death

We have shown that lgi1b morphants have smaller brains (Figure 3). Previously, we demonstrated that a reduced brain size in lgi1a morphants was due to increased apoptosis, mostly in the forebrain [Teng et al 2010]. Since developmental brain defects could be due to several underlying mechanisms, such as increased cell death or reduced proliferation, we first examined apoptosis in the lgi1b-deficient embryos using acridine orange staining. During early embryogenesis (from 24 to 72 hpf) lgi1b morphants showed increased apoptosis throughout the brain after 36 and 48 hpf, compared with wild-type embryos and mismatch morphants, where cell death was negligible (Figure 4B and 4E). Interestingly, at 72 hpf, significantly increased cellular apoptosis was seen, specifically in the forebrain of lgi1b morphants (Figure 4C), compared to mid- and hindbrain regions. These observations were confirmed using TUNEL assays (Figure 4D). In mRNA rescue assay, increased apoptosis levels were significantly reduced throughout the brain compared to lgi1b morphants (Figure 4E). To examine cell proliferation rates we used BrdU incorporation where, at 48 hpf, there was no significant difference between the MO-E2 and MO-E2mis injected embryos (Figure S3). Thus, loss of lgi1b does not appear to affect cell proliferation but has a significant effect on apoptosis during early brain development.

### Lgi1b morphants show increased sensitivity to PTZ treatment

**Figure 5:**
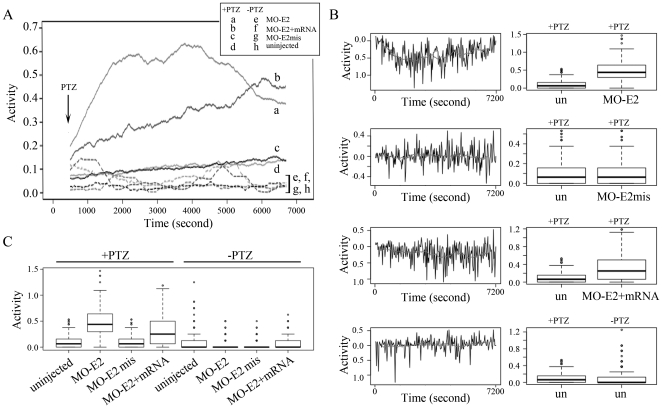
Synergistic PTZ induction of seizure-like behavior in lgi1b morphants. (A) Longitudinal plot of average activity of 72 hpf embryos over a 2 hour period using Viewpoint modeling software time series plots and a custom smoothing algorithm described by Teng et al (2010). Dotted lines depict the four classes of fish without PTZ treatment (e, f, g, h). Solid lines depict morphants and controls according to key (above). In the absence of PTZ (dotted lines) fish activity (n = 96 for each group) is relatively low in the 2 ng morphants and wild type fish. Upon treatment with PTZ (solid lines) average activity increases significantly in MO-E2 morphants (a) but not in the mismatch morphants (c) or wild type fish (d). Morphants co-injected with rescue lgi1b mRNA show a significant reduction in activity (b) compared with the MO-E3 morphants. (B) Box plots generated in R statistical software showing the distribution of average fish activity over time for different experimental conditions as shown. (C) Comparison of the longitudinal differences in mean values between experimental groups. The red trace follows the moving averages longitudinally (left). Box plots of the same data are shown (right).

To analyze behavioral changes as a result of *lgi1* knockdown, we have developed a statistical approach to evaluate motor activity behaviors of individual fish over a fixed time period (2 h) using the Zebralab monitoring system and Viewpoint software [22]. This analysis takes into account not only swimming activity (tracking) but also localized hyperactivity in the fish embryos to account for all activity patterns. There was no obvious seizure-like behavior in *lgi1b* morphants at 72 hpf, as seen in lgi1a morphants [Teng et al 2010] or PTZ treated fish [23]. To determine whether *lgi1b* morphants were also sensitized to PTZ-induced seizures, we treated low dose morphants (2 ng) with 2.5 mM PTZ, which was previously shown to be the minimal concentration that could be used to induce significant changes in swimming behavior in 3 dpf, uninjected fish [Teng et al 2010]. For each experiment, six different groups were analyzed; uninjected wild type controls, MO-E2mis morphant controls, and MO-E2 morphants; each being treated with either PTZ or control media. After a 30 minute ‘baseline’ observational period, either 2.5 mM PTZ or control media was added to all wells and overall activity was monitored over the next 2 hours. The resulting data was processed using MatLab modeling software and analyzed using a custom biostatistical approach that allowed pooling data from independent experiments [22]. As shown in Figure 5, wild-type and mismatch morphants (MO-E2mis) showed only a mild increase in hyperactivity following low dose PTZ treatment. In contrast, a highly significant increase in hyperactivity was seen in the MO-E2 morphants (Figure 5). This increased hyperactivity was rescued to some extent, although not eliminated, in the mRNA rescue experiment (Figure 5). Thus, while knockdown of *lgi1b* could not induce overt seizure-like behavior, morphants are sensitized to drug-induced seizure-like behavior.

### Analysis of c-fos expression in lgi1 morphants

**Figure 6:**
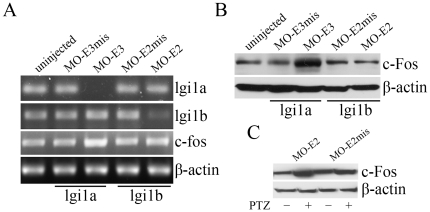
Analysis of c-fos expression as a marker for seizure-like behavior. Increased expression of c-fos in lgi1a (MO-E3), but not lgi1b (MO-E2), morphants compared with corresponding mismatch (mis) MO treatment. RNA and protein extracts from 3 dpf embryos injected with 3 ng of the indicated morpholinos or wildtype were used for semi-qRT-PCR (A) and western blot assays (B), respectively. When lgi1 morphants (MO-E2) were treated with PTZ, an increase in c-fos expression was observed. No increase in c-fos was seen when mismatch morphants (MO-E3mis) were treated with PTZ.

It has been demonstrated previously [30]–[31] that seizure-like behavior is accompanied by an increase in expression levels of c-fos in neurons. RT-PCR analysis of 3 dpf embryos injected with 3 ng MO (Figure 6A) demonstrated an increase in c-fos expression in lgi1a morphants (MO-E3) that experience seizure-like behavior, compared with mismatch control morphants (MO-E3mis). In contrast, c-fos levels in lgi1b morphants (MO-E2) did not differ significantly from those seen in the corresponding mismatch control (MO-E2mis). These changes were more pronounced using western blot analysis of c-fos protein levels (Figure 6B). When the lgi1b morphants were treated with PTZ the increased activity observed in these fish was accompanied by an increase in c-fos expression which was not seen in the MO-E2mis morphants treated with PTZ (Figure 6C).

### Lgi1a/lgi1b double morphants show compound phenotypes and increased mortality

**Figure 7:**
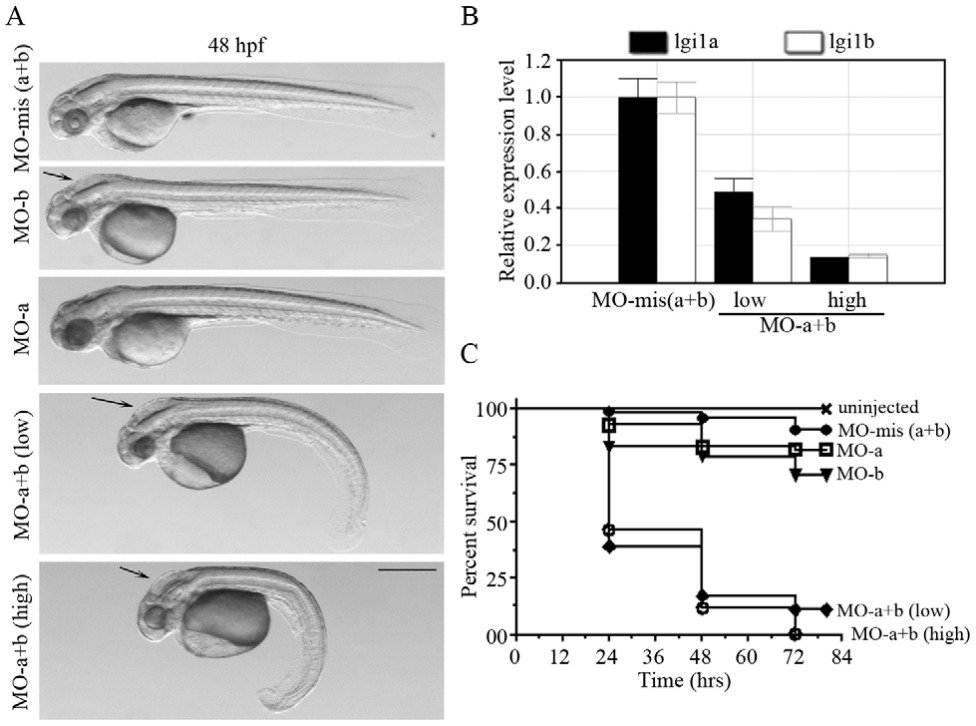
Analysis of lgi1a and lgi1b double morphants. Coincident knockdown of lgi1a and lgi1b results in enhanced phenotype and high mortality rates compared with the single morphants. In (A) 2 ng morphants for lgi1a shows a macroscopically normal phenotype, which is similar to lgi1b morphants, other than the presence of hydrocephalus (arrow). The double mismatch morphant (3 ng of each MO) also appears normal. When the double morphant was analyzed using low dose MO (2 ng of each) small eyes and head together with hydrocephalus (arrow) are observed as well as a curved tail phenotype not seen in single morphants. At high MO doses (3 ng) the curved tail phenotype is enhanced. (B) Quantitative PCR analysis shows that combined treatment with mismatch MO has no effect on either lgi1a or lgi1b mRNA whereas combined low dose (2 ng) treatment results in a 35–50% reduction in mRNAs from the two genes which is even further reduced after high dose (3 ng) treatment. In (C) the mortality in both low dose and high dose combined treatment is significantly different than in the single morphants or combined mismatch morphants. After 48–72 hpf the mortality is even further increased.

The knockdown of lgi1a [22] led to the development of a seizure-like behavior as well as developmental abnormalities involving the brain and eyes and, at high doses of morpholino, to abnormalities of the tail. The lgi1b morphants also showed abnormalities of the development of the eye and brain but did not show abnormalities of the tail and did not show seizure-like behavior, although both morphants were sensitized to PTZ-induced hyperactivity. The unique feature of the lgi1b morphant was the pronounced hydrocephalus. To determine the effects of knocking down both lgi1 paralogs, we created dual morphants by injecting embryos with both the MO-E3 and MO-E2 morpholinos for the lgi1a and lgi1b genes respectively. Since the compound morphants were likely to experience more severe phenotypes, reflecting the importance of both genes for normal development, we created morphants using high (3 ng) and low (2 ng) concentrations of the morpholinos (figure 7) and compared them to morphants created using the equivalent concentrations of the mismatch morpholinos. Compared with the single morphants, the compound morphants showed a much higher incidence of premature death with ∼50% dying after 24 hfp and >75% mortality after 48 hpf. The high dose morphants showed slightly greater mortality compared with the low dose. The single morphants, over the 72 hpf period, only showed mortality of ∼20% (figure 7C). As we have reported throughout this series of experiments the mismatch morphants do not show any developmental abnormalities even at high dose of MO. At a low dose (2 ng) lgi1a and lgi1b morphants do not show abnormalities of the tail (figure 7A), which is correlated with a proportional knockdown of the respective mRNAs in the enbryos (figure 7B). In contrast, the compound morphants show significant tail deformity in addition to smaller eyes and head size (Figure 7A). The high-dose, compound morphants show ever more dramatic developmental abnormalities than the low dose morphants (figure 7A). One of the striking observations in the development of the compound morphants was their inability to escape from the chorion, which precluded behavioral analysis as described in figure 5. For those rare embryos that survived beyond 48 hours, when we manually removed the chorion, once freed, these embryos appeared to show a hyperactivity not seen in mismatch morphants, which suggests a similar phenotype to that seen in lgi1a morphants. In addition, the compound morphants at low or high dose demonstrated the hydrocephalus seen in the lgi1b morphants (Figure 7A). These data suggest firstly, that specific phenotypes related to knockdown of the individual lgi1 genes are retained in the compound morphants and that lower doses of the individual MOs can produce the more extreme phenotypes seen only with high doses of the individual MOs. The high mortality rate and early onset of death in the embryos precluded many of the behavioral studies but supports an important role for the lgi1 genes in development.

## Discussion

Duplication of the zebrafish genome allows subfunctionalization of the paralogs. This appears to be the case for the *lgi1* genes. Lgi1a morphants demonstrate seizure-like behavior within the 3–4 ng range, whereas the lgi1b morphants do not. Inactivation of either paralog, however, predisposes to PTZ induced hyperactivity using low dose (2 ng) MO treatments, which indicates a potentially common response to this epilepsy inducing drug. It has also been shown that mice with heterozygous inactivation of Lgi1 are hyper sensitive to PTZ induced seizures [17]. The lgi1b morphants show a pronounced enlargement of the ventricles, which was not seen in the lgi1a morphants. Interestingly, the Lgi1 gene was shown to be highly expressed in the choroid plexus (CP) in mice [6], which is also seen at early stages of embryonic brain development [32]. The CP lines the four ventricles and regulates fluid transport across the blood-cerebrospinal fluid (CSF) barrier, and so controls CSF volume. The expression of Lgi1, even at the earliest stages of CP development, suggests an important function for this protein in these cells throughout the lifetime of the organism, since its high expression levels are maintained in the adult CP. In fish it appears that this function may be regulated by Lgi1b.

Recently we performed an extensive survey of Lgi1 expression in the developing mouse embryo [32] and demonstrated that, at early stages of development, lgi1 expressing cells also express nestin and doublecortin, suggesting a function in neural progenitor cells. It has been shown recently that MO knockdown of zebrafish nestin produces a very similar phenotype to that seen in the lgi1b morphants [33]. Both morphants show smaller eye size and brain mass, which is attributable to increased apoptosis in both cases. The hydocephalus seen in the lgi1b morphants were also seen in the nestin morphants and neither morphant exhibits the seizure-like behavior seen in the lgi1a morphants. Nestin is an intermediate filament protein which interacts with vimentin and desmin to form part of the cellular cytoskeleton and is expressed primarily in neuroepithelial precursor cells and proliferating neural progenitor cells. Since LGI1 is a secreted protein, it is unlikely to interact directly with cytoskeletal proteins but the striking resemblance in phenotypes in the nestin and lgi1b morphants, together with the observation that Lgi1 is expressed in nestin expressing cells, suggests that the function of these two proteins may be interconnected.

The phenotypic consequences of lgi1b knockdown involve abnormal development of the brain with increased apoptosis as well as abnormal eye development, suggesting an important role for this gene in the development of these organs. This observation is supported by the expression pattern for lgi1 defined by Gu et al [27] using in situ hybridization. At 24 hours, lgi1b was expressed in presumptive telencephalic and diencephalic bands and the cranial paraxial mesenchyme By 48 hours lgi1b was expressed in the optic tectum, cerebellum and a zone of migratory neurons that originated from the rhombic lip as well as in the dorsal thalamus and the retinal ganglion layers. Generally, although some overlap with the expression of lgi1a, expression of lgi1b was dorsally restricted in the mid and hind brain which is consistent with the distribution of apoptosis seen in the lgi1b morphants. It is interesting that lgi1b expression is seen in subsets of migrating neurons since molecular [15] and developmental [32] analysis of the mammalian LGI1 gene also suggests a role in neuronal migration. Lgi1, however, is a secreted protein and it is not clear whether loss of function in morphants leads to a cell autonomous phenotype or whether the loss of protein function affects cells that would normally be responsive to its presence.

The seizure-like behavior seen in lgi1a is very different to that seen in the lgi1b morphants and we are currently investigating whether there is an underlying electrophysiological basis of this difference. Both lgi1 morphants, however, show a sensitization to PTZ-induced hyperactivity. It was demonstrated previously that the c-fos gene can act as a useful marker for elevated levels of neuronal activity following seizure [34]. We have now shown that increased fos expression is seen in the lgi1a morphants, but not the lgi1b morphants unless they were treated with PTZ. These data correlate well with the behavioral phenotype and provide support for the interpretation that the seizure-like hyperactivity seen in the lgi1a morphants is similar to mammalian seizures. In addition, the absence of seizure-like behavior in the lgi1b morphants, even following high dose MO treatment, together with no change in fos expression, further demonstrates the distinct functions of these genes.

## Supporting Information

Figure S1 **48 hpf lgi1b morphants (lateral view) injected with high dose (4 ng) MO-E2 show abnormal developmental phenotypes with more severe hydrocephalus (arrow), small eyes and curved tails (arrow head).** Embryos injected with the MO-E2mis did not show these phenotypes. Scale bar: 500 µm.
(TIF)

Figure S2 **(A) RT-PCR analysis shows that mRNA levels in lgi1b morphants (2 ng) recover after 4 dpf. (B) Severe hydrocephalus (arrow), heart edema (arrow head) and smaller eyes were still observed at 5 dpf in lgi1b morphants (lateral view).** Scale bar: 500 µm.
(TIF)

Figure S3 **Immunofluorescence analysis of BrdU incorporation in MO-E2 vs. MO-E2mis injected embryos at 48 hpf.** Comparison of proliferating cells in forebrain regions of lgi1b knockdown morphants shows no significant difference from that in control morphants. Scale bar: 50 µm.
(TIF)
