# Supplementary figures and images for: Loss of Zebrafish lgi1b Leads to Hydrocephalus and Sensitization to Pentylenetetrazol Induced Seizure-Like Behavior
Source: PLoS One. 2011 Sep 16;6(9):e24596. doi: 10.1371/journal.pone.0024596 (PMC3203530; doi:10.1371/journal.pone.0024596)

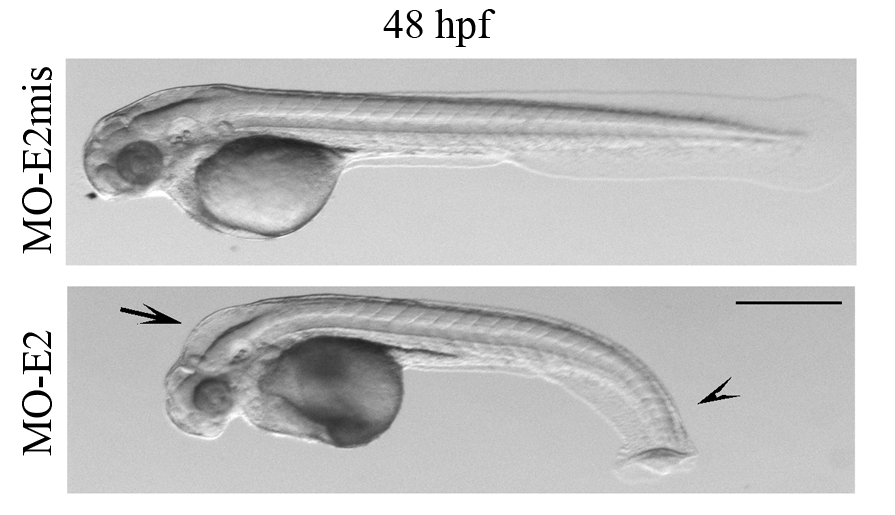

Supplement: Figure S1 — 48 hpf lgi1b morphants (lateral view) injected with high dose (4 ng) MO-E2 show abnormal developmental phenotypes with more severe hydrocephalus (arrow), small eyes and curved tails (arrow head). Embryos injected with the MO-E2mis did not show these phenotypes. Scale bar: 500 µm. (TIF) [file pone.0024596.s001.tif]

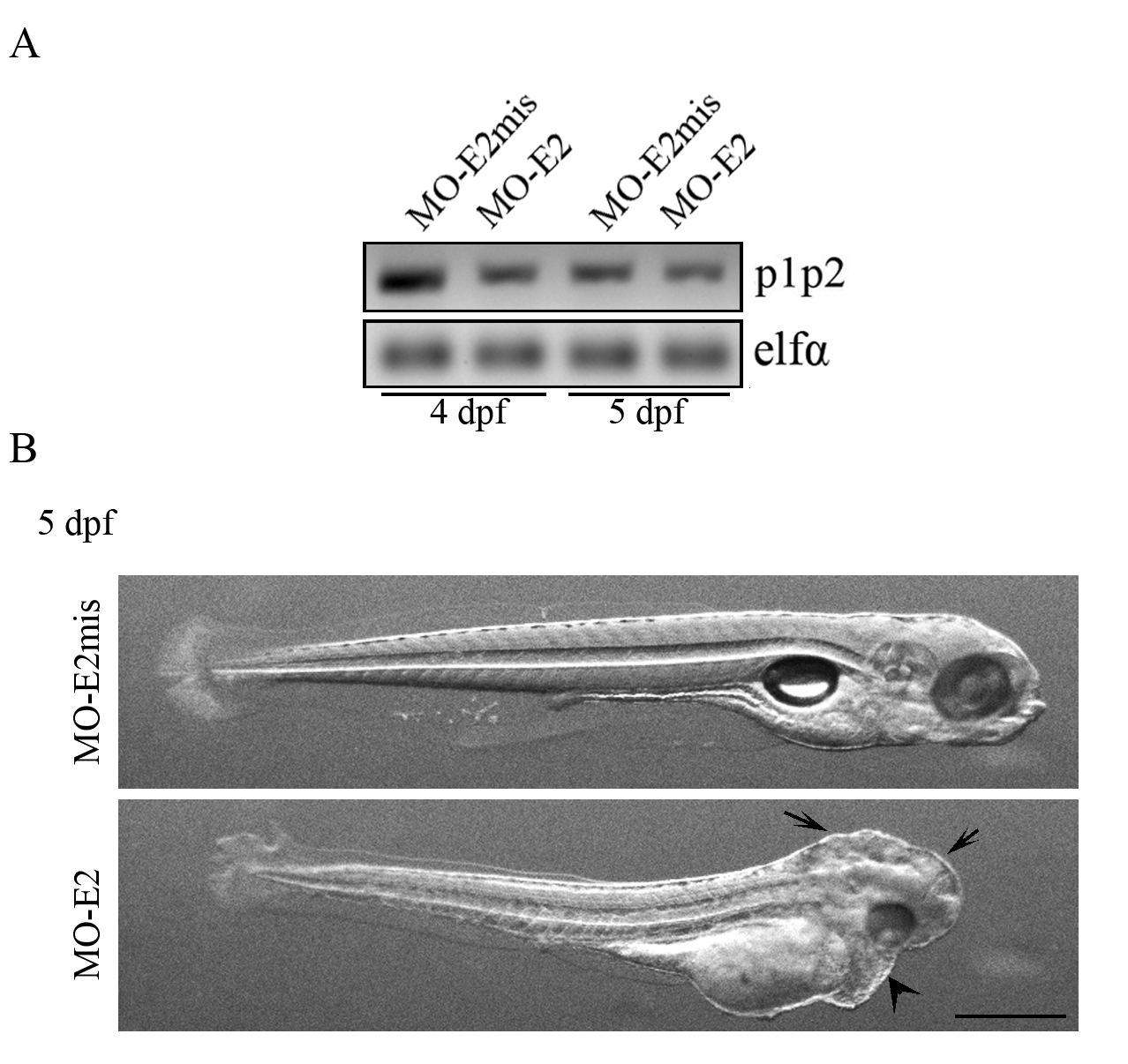

Supplement: Figure S2 — (A) RT-PCR analysis shows that mRNA levels in lgi1b morphants (2 ng) recover after 4 dpf. (B) Severe hydrocephalus (arrow), heart edema (arrow head) and smaller eyes were still observed at 5 dpf in lgi1b morphants (lateral view). Scale bar: 500 µm. (TIF) [file pone.0024596.s002.tif]

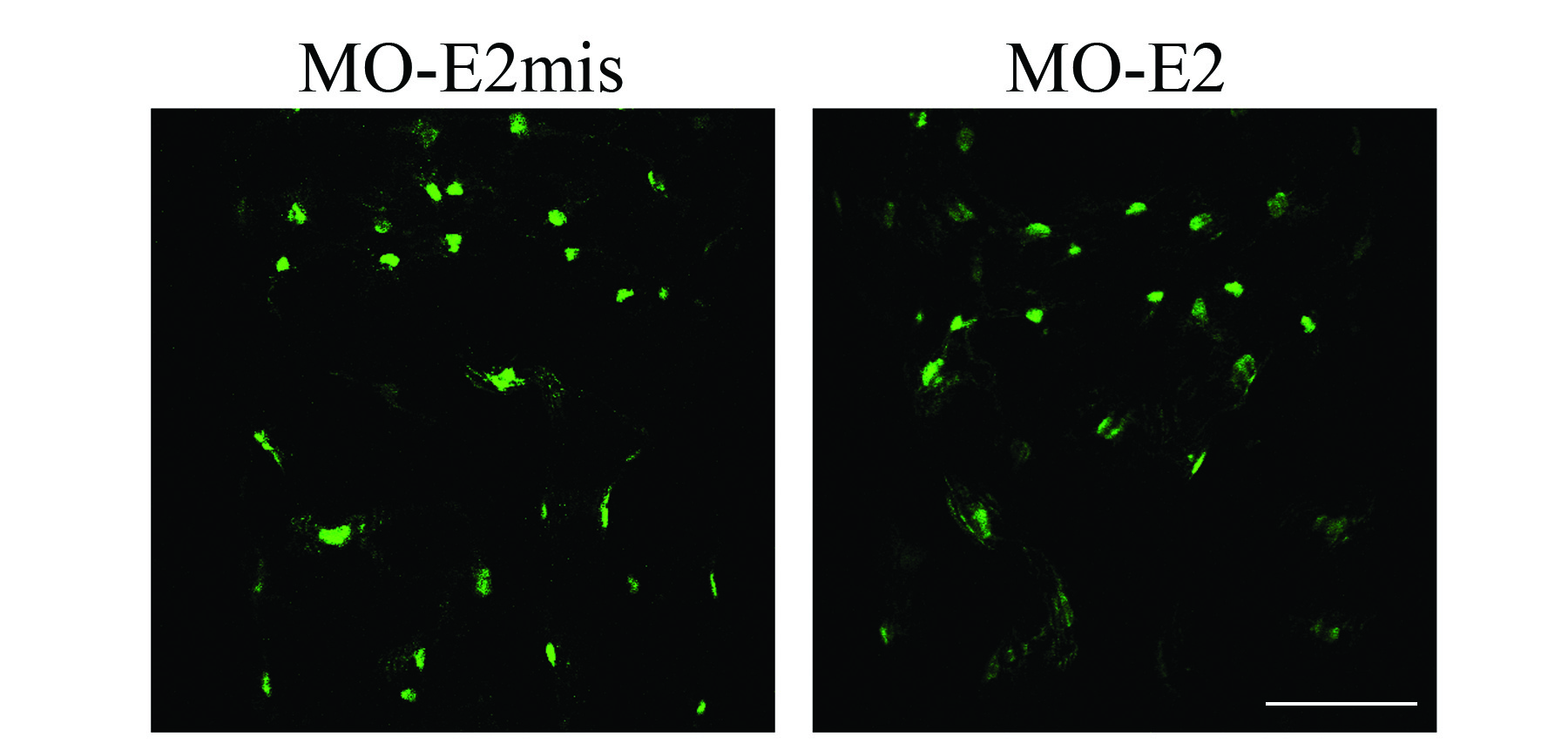

Supplement: Figure S3 — Immunofluorescence analysis of BrdU incorporation in MO-E2 vs. MO-E2mis injected embryos at 48 hpf. Comparison of proliferating cells in forebrain regions of lgi1b knockdown morphants shows no significant difference from that in control morphants. Scale bar: 50 µm. (TIF) [file pone.0024596.s003.tif]
